# The relationship between teacher leadership style and learning engagement of English major students—the mediating role of academic self-efficacy

**DOI:** 10.3389/fpsyg.2026.1781211

**Published:** 2026-05-14

**Authors:** Chunyu Song, Baitong Zhu, Yanyan Huang, Dandan Xie

**Affiliations:** 1 School of Languages and Cultures, Youjiang Medical University for Nationalities, Baise, Guangxi, China; 2 Life Science and Clinical Medicine Research Center, Affiliated Hospital of Youjiang Medical University for Nationalities, Baise, Guangxi, China; 3 Damiao Middle School of Songshan District, Chifeng, China; 4 Guangxi International Business Vocational College, Nanning, Guangxi, China

**Keywords:** academic self-efficacy, English major students, Learning engagement, mediating effect, teacher leadership

## Abstract

**Introduction:**

Teacher leadership is an important factor that likely influences students’ learning engagement. However, teacher leadership has received less attention from researchers than other potential causes of learning engagement. Previous research has focused mainly on the perspectives of principals (i.e., academic leaders) and teachers. Student-perceived teacher leadership has received comparatively less empirical attention, or conducted in-depth research on the relationships between this factor and students’ academic self-efficacy and learning engagement.

**Methods:**

This study explores the relationships among teacher leadership and English major students’ academic self-efficacy and learning engagement in the foreign language context. A questionnaire was completed by 591 English major students at three common colleges via a random sampling method.

**Results:**

The results showed teacher leadership, academic self-efficacy, and learning engagement were all positively correlated with each other. Teacher leadership had significant impacts on both academic self-efficacy and learning engagement, with academic self-efficacy mediating the relationship between teacher leadership and learning engagement.

**Discussion:**

Therefore, enhancing the teacher leadership of college teachers and promoting students’ academic self-efficacy can effectively boost learning engagement among English major students and promote a positive academic atmosphere in foreign language education of colleges.

## 1 Introduction

In recent years, positive psychology has been increasingly applied in educational research and practice by scholars both domestically and internationally. As a key indicator of students’ learning quality and a crucial academic experience in higher education, learning engagement has consistently attracted scholarly attention in the fields of psychology and education. A growing body of research has sought to identify the factors that influence students’ learning engagement, generally categorizing them into four main domains: individual, family, peer, and teacher-related factors.

With regard to individual factors, academic self-efficacy has been widely recognized as a robust predictor of learning engagement. Voelkl (1997) demonstrated a positive association between students’ confidence in completing academic tasks and their level of engagement, suggesting that students with stronger self-efficacy are more likely to invest sustained effort in learning activities.

Teacher-related factors have also been extensively examined in previous research. Wang (2020) classified teacher-related influences into instructional behavior, students’ background characteristics, curriculum resources and course management, as well as course goals and understanding. Her empirical findings indicated that teachers’ instructional behaviors—such as perceived support, teaching style, and the quality of teacher–student relationships—exert a particularly strong influence on students’ learning engagement. More recent studies have further expanded this line of inquiry by examining teacher leadership or related leadership constructs and their associations with student engagement in higher education contexts (Assefa et al., 2025; Kibret and Berhanu, 2025; Wang S. et al., 2024). A study emphasized the importance of school climate, especially of the relationship and educational style of teachers on the commitment and school attendance of students (Sorrenti et al., 2024). Positive teacher-student relationships significantly promote students’ academic success by enhancing their sense of emotional support, self-efficacy, and classroom engagement (Camp, 2011).

English, as a global language, constitutes a fundamental competency for university students in the context of globalization and the vision of a community with a shared future for mankind. For English major students, language learning is not only an academic requirement but also a critical means of engaging with the international community. However, learning a second language often involves additional psychological and emotional challenges, including language anxiety and stress arising from linguistic barriers and cultural differences (Han et al., 2022). As a result, English major students are required to invest considerable time and cognitive effort, making their learning engagement a particularly important issue for investigation.

Although existing studies have increasingly acknowledged the role of teacher leadership in shaping student engagement, several limitations remain. First, much of the existing literature has focused on general higher education contexts or has emphasized leadership at the institutional level, such as principals or administrators, rather than examining teacher leadership as perceived by students in specific disciplinary settings. Second, relatively fewer studies have simultaneously incorporated teacher leadership style as an independent variable and academic self-efficacy as a mediating psychological mechanism when examining learning engagement, particularly from the perspective of English major students in language education contexts.

Therefore, the present study seeks to extend existing scholarship by focusing on a specific student group—English majors—and by integrating both teacher-related and individual psychological factors into a unified analytical framework. Specifically, this study examines the relationships among teacher leadership style, academic self-efficacy, and learning engagement, with the aim of uncovering the underlying mechanisms through which teacher leadership influences students’ engagement in language learning. By adopting the student perspective and situating the analysis within the field of foreign language education, this study contributes to educational psychology research and provides a theoretical basis for universities to develop targeted strategies to enhance English major students’ learning engagement.

## 2 Theoretical review and research hypotheses

### 2.1 Teacher leadership style and learning engagement

A widely accepted and extensively utilized conceptualization of learning engagement is the multidimensional framework synthesized by Fredricks et al. (2004) and further elaborated by Fredricks and McColskey (2012), based on a substantial body of empirical research in school and higher education contexts. This framework conceptualizes learning engagement as comprising three interrelated dimensions: cognitive, emotional, and behavioral engagement. The cognitive dimension refers to students’ psychological investment in learning, including effort, persistence, and the use of deep learning strategies; emotional engagement reflects students’ interest, enjoyment, and value attached to learning tasks; and behavioral engagement captures observable participation in academic activities and adherence to institutional norms. Subsequent empirical studies across different cultural and educational contexts have consistently supported the reliability and validity of this tripartite model, demonstrating its applicability in diverse national and disciplinary settings (Appleton et al., 2008; Christenson et al., 2012; Reschly and Christenson, 2012). Cross-cultural research has further indicated that the cognitive–emotional–behavioral structure of learning engagement is robust among students from different cultural backgrounds, including East Asian and Western contexts (Lam et al., 2014; Li and Lerner, 2013). Owing to its strong theoretical grounding and empirical support, this multidimensional definition has gained broad international recognition and has been widely adopted in studies of student engagement in higher education. Accordingly, the present study employs this established framework to conceptualize English major students’ learning engagement.

A growing body of research suggests that college students’ learning engagement is shaped by a complex interplay of individual characteristics, learning environments, curricular structures, and teacher-related factors. Among these, teacher leadership has emerged as an increasingly salient construct. Teacher leadership broadly refers to teachers’ capacity to influence students, colleagues, and institutional practices through professional expertise, collaborative actions, and pedagogical decision-making, thereby contributing to both student learning and school improvement (Bascia, 1997; Sun, 2018; York-Barr and Duke, 2004). Unlike formal administrative leadership, teacher leadership emphasizes teachers’ informal influence within instructional and professional contexts. Existing scholarship on teacher leadership has developed along several major thematic lines. Prior studies have examined teacher leadership in relation to teacher professionalism and identity development (Muijs and Harris, 2006), distributed and shared leadership within schools (Harris, 2008), teachers’ professional growth and mentoring roles (York-Barr and Duke, 2004), as well as educational reform and school improvement processes (Harris and Muijs, 2005). More recent empirical research has begun to link teacher leadership behaviors directly to student-level outcomes, including academic achievement and learning engagement, particularly in higher education settings (Assefa et al., 2025; Kibret and Berhanu, 2025; Wang X. et al., 2024). These studies suggest that teacher leadership constitutes an important teacher-related factor that merits closer examination in engagement research.

This study extends prior research by focusing on student-perceived teacher leadership among English major students, emphasizing how teacher behaviors influence students’ engagement through both direct and indirect pathways, rather than solely focusing on teacher-reported leadership.

Within the field of foreign language education, teacher leadership has gradually attracted scholarly attention. Language teachers often play a dual role as instructional leaders and curriculum mediators, shaping learning goals, pedagogical practices, and academic expectations within their disciplinary communities. Research indicates that effective teacher leadership in language education can enhance instructional coherence, foster supportive classroom climates, and promote students’ sustained engagement in language learning tasks (Han et al., 2022; Wang X. et al., 2024). Nevertheless, compared with general higher education contexts, empirical studies focusing specifically on teacher leadership as perceived by English major students remain relatively limited. To conceptualize teacher leadership styles, this study adopts the Charismatic, Ideological, and Pragmatic (CIP) leadership model proposed by Mumford et al. (2009), which has been widely applied in leadership research. Charismatic leadership emphasizes vision articulation, personal influence, and emotional appeal, enabling teachers to inspire students’ identification with learning goals. Ideological leadership is grounded in deeply held values and moral beliefs, stressing purpose, ethics, and long-term commitment to learning. Pragmatic leadership focuses on problem-solving, goal attainment, and evidence-based instructional practices that help students manage learning tasks effectively. Empirical research has demonstrated that different leadership styles exert distinct influences on student engagement and related academic outcomes. Charismatic teacher leadership has been associated with higher levels of classroom participation and enthusiasm, particularly in interactive learning environments (Bolkan and Goodboy, 2011; Griffith et al., 2015). Pragmatic leadership facilitates learning engagement by clarifying expectations, structuring tasks, and providing timely feedback, thereby enhancing students’ focus and perceived competence (Hunter S. T. et al., 2009; Hunter S. T. et al., 2011). Ideological leadership contributes to deeper forms of engagement by fostering students’ sense of meaning, ethical awareness, and intrinsic motivation for learning (Eyal et al., 2020; Shin and Cho, 2017). The influence of teacher leadership on learning engagement operates not only through direct instructional practices but also through indirect pathways, such as shaping classroom climate, promoting emotional identification, and supporting students’ self-regulatory processes. Bryk et al. (2010) highlighted that effective teacher leadership contributes to a supportive learning environment, which enhances students’ emotional and behavioral engagement and, ultimately, academic performance.

By considering the unique learning challenges of English major students, including second language acquisition demands and language anxiety, this study provides a discipline-specific perspective on how teacher leadership shapes engagement. Hypothesis 1 is proposed: Teacher leadership has a significant positive impact on English major students’ learning engagement.

### 2.2 Teacher leadership style and academic self-efficacy

Academic self-efficacy refers to an individual’s belief in their capability to successfully complete academic tasks and achieve learning goals (Bandura, 1997). It is a core concept derived from social cognitive theory and has been widely recognized as a critical predictor of students’ motivation, learning behavior, and academic performance. Many researchers have provided conceptual definitions of academic self-efficacy. Sander (2006) defined academic self-efficacy as students’ evaluation of their likelihood of success in completing academic tasks. When academic self-efficacy is high, individuals are more likely to expect themselves to work harder to achieve learning goals. Wang and Carrie (2008) viewed academic self-efficacy as students’ belief in their ability to successfully perform specific academic tasks during the learning process. In China, scholar Liang (2000) defined academic self-efficacy as students’ evaluation and judgment of their ability to successfully complete academic tasks, achieve excellent academic performance, and avoid academic failure. It also includes the students’ assessment of whether they can meet academic requirements through learning methods that suit their personal characteristics. In recent years, a growing number of domestic studies have adopted Liang Yusong’s definition. This study also adopts Liang Yusong’s conceptualization of academic self-efficacy.

There is limited research on the relationship between teacher leadership style and academic self-efficacy. However, the few existing studies suggest that teacher leadership influences students’ academic psychological development from multiple dimensions—particularly the development of academic self-efficacy—by shaping teacher-student interactions, classroom climate, and students’ sense of belonging. Schunk and Pajares (2005) pointed out that the development of self-efficacy is influenced by various factors, including mastery experiences, vicarious experiences, emotional states, and verbal persuasion. Teacher leadership behaviors serve as key external inputs for these sources. Additionally, Liu and Hu (2021), through structural equation modeling, confirmed that teacher leadership indirectly influences the development of students’ self-efficacy by enhancing emotional support and learning engagement. Charismatic teacher leadership emphasizes the teacher’s personal charisma, expressive motivation, and emotional contagion. Such teachers act as role models and transmit positive emotions, stimulating students’ intrinsic motivation and confidence in learning, thereby strengthening their belief in their ability to complete academic tasks (Conger and Kanungo, 1987). Pragmatic leadership emphasizes clear goals, instructional process management, and continuous feedback mechanisms. In the classroom, these teachers establish clear learning goals, provide phased guidance, and offer timely feedback, helping students form predictable and achievable learning paths, which enhances their sense of control and accomplishment (Bass and Avolio, 1994). Ideological leadership focuses on guiding students through the instillation of values, belief systems, and educational ideals. It emphasizes the social significance of learning and the mission of personal growth. Under this style, teachers’ leadership behaviors encourage students to integrate learning tasks with their self-identity, strengthen expectations for personal development, and thus enhance their academic self-efficacy.

By linking teacher leadership to academic self-efficacy in English major students, the study examines how leadership behaviors influence students’ internal belief systems, bridging classroom practices and psychological mechanisms. Hypothesis 2 is proposed: Teacher leadership has a significant positive impact on the academic self-efficacy of English major students.

### 2.3 Academic self-efficacy and learning engagement

Academic self-efficacy has long been a central focus in the fields of psychology and education, as it plays a significant role in students’ learning. In addition to effectively predicting individuals’ academic emotions, academic self-efficacy also positively predicts students’ learning engagement and academic achievement, specifically, academic self-efficacy influences learning engagement by affecting whether students are willing to participate in learning tasks, as well as the duration of their effort and persistence during the learning process (Shoulders and Krei, 2015). Students with high academic self-efficacy tend to choose challenging tasks, exhibit greater self-confidence, and are more willing to persevere when facing difficulties. They are more likely to experience positive emotions, engage more actively in coursework, and achieve better academic results (Uçar and Sungur, 2017). In contrast, students with low academic self-efficacy are more likely to avoid difficult learning tasks and show less engagement in class compared to those with higher self-efficacy (Martin and Rimm-Kaufman, 2015). This avoidance behavior not only reduces their classroom engagement but also affects their psychological state and learning outcomes. Xu et al. (2021) pointed out that effectively enhancing students’ academic self-efficacy can significantly increase their learning engagement and bring about positive emotional experiences. Students with high self-efficacy are more likely to set challenging goals and actively adjust their learning strategies, thereby demonstrating higher levels of learning engagement. A study among vocational students demonstrated that academic self-efficacy mediated the relationship between perceived teacher leadership and students’ deep learning engagement (Lu and Wang, 2025).

This study further examines academic self-efficacy as a mediator between teacher leadership and learning engagement specifically among English major students, providing empirical evidence for the mechanism through which leadership affects engagement in language learning contexts.

In summary, academic self-efficacy is a critical factor influencing students’ learning engagement. It affects whether individuals choose to participate in a learning activity, as well as the level of effort and persistence they demonstrate. Hypothesis 3 is proposed: Academic self-efficacy has a positive impact on the learning engagement of English major students and mediates the relationship between teacher leadership and learning engagement.

## 3 Materials and methods

### 3.1 Research participants

We selected 17 classes from three common universities at random through the university administration. The questionnaires were collected on site via a random sampling method. A total of 630 questionnaires were distributed to the English major students, of which 591 were valid, yielding an effective response rate of 93.81%. Among the included students, Males accounted for 58.88% (*n* = 348), and females for 41.12% (*n* = 243). Overall, the gender ratio was relatively balanced. The grade and place of residence ratios are generally balanced, indicating a near-equal representation and suggesting the sample was representative. These respondents participated in this study anonymously and voluntarily, and were informed of the risks and benefits of participation. It was clearly stated that they had the right to withdraw from the study at any time without any consequences. There were not specific factors that might influence the generalizability of the findings.

### 3.2 Research instruments

The questionnaire utilized in this study comprised three scales: teacher leadership style, academic self-efficacy, and learning engagement. All questionnaires were administered in Chinese and each rated on a 5-point Likert scale (1 = strongly disagree, 2 = disagree, 3 = uncertain, 4 = agree, 5 = strongly agree). The scales were categorized based on score ranges as follows: 1–2.33 (low), 2.34–3.66 (moderate), and 3.67–5 (high). Since the questionnaire contained a scale originally developed in English, a double back-translation method was employed prior to administration to ensure consistency between the English and Chinese versions. Specifically, one translator first translated the scales from English into Chinese. Once this translation was completed, a second translator independently translated the Chinese version back into English. Finally, two additional validators compared the English and Chinese versions to confirm their consistency.

#### 3.2.1 Teacher leadership style scale

This study adopted the Teacher Leadership Style Scale developed by Tsai (2017). The scale consists of three dimensions: charismatic leadership (14 items), pragmatic leadership (11 items), and ideological leadership (4 items). Charismatic leadership involves a future-oriented vision that emotionally inspires and attracts followers. Ideological leadership fosters unity among group members through shared beliefs. Unlike charismatic and ideological leadership, pragmatic leadership emphasizes addressing current issues to drive group motivation. In this study, the Cronbach’s α coefficient for this scale was 0.909, with all factor loading exceeding 0.5, KMO value was 0.927, demonstrating strong reliability and validity.

#### 3.2.2 Academic self-efficacy scale

This study adopted the academic self-efficacy scale compiled by Chinese scholars Liang (2000). The questionnaire consists of two dimensions: self-efficacy of learning ability (12 items) and self-efficacy of learning behavior (12 items). The 5-point Likert score is adopted. The higher the score, the higher the level of academic self-efficacy of the subjects (item 14,16,17 and 20 are reverse scoring items). In this study, the Cronbach’s α coefficient for this scale was 0.868, with all factor loading exceeding 0.5, KMO value was 0.908, demonstrating strong reliability and validity.

#### 3.2.3 Learning engagement scale

Compiled by Liao (2011), the scale consists of three dimensions: behavioral engagement (6 items), cognitive engagement (7 items), and emotional engagement (7 items). Behavioral engagement refers to students’ specific behaviors during the learning process, such as time management, learning strategies, and participation in learning activities. Cognitive engagement pertains to the mental activities students invest in learning, including attention, thinking, and comprehension. Emotional engagement describes students’ emotional responses to learning activities, such as interest, satisfaction, and emotional experiences. In this study, the Cronbach’s α coefficient for this scale was 0.905, with all factor loading exceeding 0.5, KMO value was 0.924, demonstrating strong reliability and validity.

### 3.3 Data processing

The data in this study were analyzed using SPSS 28.0 and AMOS 28.0. First, Common method bias and Cronbach’s α coefficients were calculated to assess the reliability of each scale and examine internal consistency. Second, Pearson correlation analysis was conducted to explore the relationships among variables, providing preliminary evidence for subsequent hypothesis testing. Third, confirmatory factor analysis (CFA) was performed to evaluate the reliability and validity of the measurement model, including composite reliability (CR), average variance extracted (AVE), and discriminant validity. Next, a structural equation model (SEM) was constructed to test the hypothesized relationships among variables. Parameters were estimated using the maximum likelihood (ML) method, and model fit was assessed using multiple fit indices, including χ^2^/df, GFI, AGFI, RMR, CFI, NFI, RFI, TLI, and IFI. Finally, the mediating effect of academic self-efficacy between teacher leadership and learning engagement was examined using the Bootstrap method, with 5,000 resamples and a 95% confidence interval.

## 4 Results

### 4.1 Demographic characteristics

A total of 591 valid questionnaires were collected in this study. Descriptive statistics were conducted on participants’ gender, academic year, and family residence. In terms of gender distribution, 58.88% were male (*n* = 348) and 41.12% were female (*n* = 243), indicating a relatively balanced gender ratio. Regarding academic year, the largest proportion of participants were sophomores (*n* = 185, 31.30%), followed by juniors (*n* = 142, 24.03%), freshmen (*n* = 133, 22.50%), and seniors (*n* = 131, 22.17%), reflecting a generally balanced distribution. As for family residence, 285 participants (48.22%) were from urban areas and 306 (51.78%) from rural areas, indicating a near-equal representation and suggesting the sample was representative.

### 4.2 Common method bias

This study used SPSS 28.0 to test for common method bias, employing Harman’s single-factor test through exploratory factor analysis. The results showed in Table 1 indicated that eight factors with eigenvalues greater than 1 were extracted before rotation, and the variance explained by the first common factor was 25.021%, well below the 40% threshold. This indicates that there is no serious common method bias in the data of this study.

**TABLE 1:** Total variance explained.

Factor	Initial eigenvalues			Extraction sums of squared loadings			Rotation sums of squared loadings		
	Total	% of variance	Cumulative %	Total	% of variance	Cumulative %	Total	% of variance	Cumulative %
1	17.765	25.021	25.021	17.765	25.021	25.021	8.524	12.006	12.006
2	8.384	11.809	36.830	8.384	11.809	36.830	7.147	10.067	22.072
3	6.088	8.574	45.404	6.088	8.574	45.404	6.724	9.470	31.542
4	3.435	4.838	50.241	3.435	4.838	50.241	6.523	9.188	40.730
5	2.987	4.207	54.449	2.987	4.207	54.449	4.456	6.277	47.007
6	2.001	2.818	57.267	2.001	2.818	57.267	4.419	6.224	53.231
7	1.785	2.514	59.781	1.785	2.514	59.781	3.926	5.530	58.761
8	1.603	2.258	62.039	1.603	2.258	62.039	2.327	3.278	62.039

### 4.3 Reliability analysis

This study used SPSS 28.0 to assess the reliability of each scale, employing Cronbach’s α coefficients to evaluate internal consistency. The results in Table 2 indicated that all variables had Cronbach’s α values above 0.85, demonstrating good internal consistency. Specifically, among the three dimensions of teacher leadership, charismatic leadership (14 items, α = 0.942) exhibited the highest reliability, pragmatic leadership (11 items, α = 0.929) ranked second, and ideological leadership (4 items, α = 0.850) showed slightly lower but still acceptable reliability. The dimensions of learning engagement also demonstrated excellent reliability, with behavioral engagement (7 items, α = 0.901), cognitive engagement (6 items, α = 0.900), and emotional engagement (7 items, α = 0.897) all around 0.90. The two dimensions of self-efficacy likewise showed strong reliability, with self-efficacy in learning ability (11 items, α = 0.943) reaching the highest level, and self-efficacy in learning behavior (11 items, α = 0.926) performing similarly well. All variables exceeded the high-reliability threshold of 0.85, confirming the reliability and stability of the questionnaires and providing a solid data foundation for subsequent analyses.

**TABLE 2:** Cronbach reliability analysis.

Variable	Items	Sample size	Cronbach α
Charismatic leadership	14	591	0.942
Pragmatic leadership	11	591	0.929
Ideological leadership	4	591	0.850
Behavioral engagement	7	591	0.901
Cognitive engagement	6	591	0.900
Emotional engagement	7	591	0.897
Self-efficacy in learning ability	11	591	0.943
Self-efficacy in learning behaviors	11	591	0.926

### 4.4 Correlation analysis

This study used SPSS 28.0 to conduct Pearson correlation analyses among the variables. As shown in Table 3, the results indicate significant positive correlations among the core research variables, while the effects of demographic variables were relatively weak. First, teacher leadership was significantly positively correlated with learning engagement (*r* = 0.387, *p* < 0.001) and with academic self-efficacy (*r* = 0.286, *p* < 0.001), suggesting that higher levels of teacher leadership are associated with stronger student learning engagement and self-efficacy. Second, learning engagement was significantly positively correlated with academic self-efficacy (*r* = 0.346, *p* < 0.001), indicating a mutually reinforcing relationship between students’ engagement and their academic self-efficacy. Regarding demographic variables, grade level showed a weak positive correlation with teacher leadership (*r* = 0.111, *p* < 0.01) and academic self-efficacy (*r* = 0.111, *p* < 0.01), family residence was weakly negatively correlated with learning engagement (*r* = −0.083, *p* < 0.05), and gender was not significantly correlated with any of the core variables.

**TABLE 3:** Pearson correlation analysis.

Variable	Gender	Grade	Family residence	Teacher leadership	Learning engagement	Academic self-efficacy
Gender	1	1	1	1	1	1
Grade	−0.054					
Family residence	0.015	−0.081				
Teacher leadership	0.022	0.111**	−0.042			
Learning engagement	0.018	0.056	−0.083*	0.387***		
Academic self-efficacy	0.021	0.111**	0.016	0.286***	0.346***	

*p < 0.05,

**p < 0.01,

***p < 0.001.

### 4.5 Validity analysis

This study employed AMOS 28.0 to conduct confirmatory factor analysis (CFA) on the measurement model in order to examine the construct validity of the scales. As shown in Table 4, the CFA results indicate that the measurement model demonstrates a good overall fit, with all fit indices meeting the recommended thresholds. The chi-square to degrees of freedom ratio (χ^2^/df = 1.535) is well below the critical threshold of 3, indicating an excellent model fit.

**TABLE 4:** Confirmatory factor model fit.

Indicator	χ ^2^ */df*	GFI	RMR	CFI	NFI	RFI	TLI	AGFI	IFI
Reference value	< 3	> 0.9	< 0.05	> 0.9	>0.9	> 0.9	>0.9	> 0.9	>0.9
Value	1.535	0.843	0.018	0.951	0.872	0.867	0.949	0.832	0.951

Regarding absolute fit indices, the goodness-of-fit index (GFI) is 0.843, approaching the ideal standard of 0.90; the root mean square residual (RMR) is 0.018, which is far below the recommended cutoff of 0.05; and the adjusted goodness-of-fit index (AGFI) is 0.832, indicating an acceptable level of fit. The incremental fit indices also show strong performance, with values of 0.951 for the comparative fit index (CFI), 0.872 for the normed fit index (NFI), 0.867 for the relative fit index (RFI), 0.949 for the Tucker–Lewis index (TLI), and 0.951 for the incremental fit index (IFI), all exceeding or approaching the recommended value of 0.90.

This study employed AMOS 28.0 to assess the convergent validity of the latent variables. As shown in Table 5, the results of the convergent validity analysis indicate that the measurement quality of all latent constructs is satisfactory and meets the requirements of structural equation modeling. All observed variables exhibit standardized factor loadings exceeding the critical threshold of 0.60, with most item loadings above 0.70, suggesting that the observed indicators effectively represent the underlying latent constructs.

**TABLE 5:** Convergent validity.

Variable	Item	Standardized coefficient	AVE	CR
Charismatic leadership	CL1	0.728	0.547	0.944
	CL10	0.71		
	CL11	0.8		
	CL12	0.794		
	CL13	0.689		
	CL14	0.759		
	CL2	0.715		
	CL3	0.684		
	CL4	0.729		
	CL5	0.81		
	CL6	0.693		
	CL7	0.78		
	CL8	0.773		
	CL9	0.666		
Pragmatic leadership	PL1	0.789	0.551	0.931
	PL10	0.801		
	PL11	0.727		
	PL2	0.687		
	PL3	0.735		
	PL4	0.724		
	PL5	0.755		
	PL6	0.743		
	PL7	0.742		
	PL8	0.699		
	PL9	0.754		
Ideological leadership	IL1	0.799	0.592	0.853
	IL2	0.724		
	IL3	0.758		
	IL4	0.793		
Behavioral engagement	BE1	0.751	0.572	0.903
	BE2	0.776		
	BE3	0.818		
	BE4	0.729		
	BE5	0.694		
	BE6	0.77		
	BE7	0.752		
Cognitive engagement	CE1	0.815	0.6	0.9
	CE2	0.794		
	CE3	0.723		
	CE4	0.766		
	CE5	0.797		
	CE6	0.752		
Emotional engagement	EE1	0.804	0.561	0.899
	EE2	0.745		
	EE3	0.71		
	EE4	0.764		
	EE5	0.705		
	EE6	0.732		
	EE7	0.778		
Self-efficacy in learning ability	SILA1	0.799	0.605	0.944
	SILA10	0.801		
	SILA11	0.832		
	SILA2	0.829		
	SILA3	0.836		
	SILA4	0.735		
	SILA5	0.782		
	SILA6	0.802		
	SILA7	0.766		
	SILA8	0.716		
	SILA9	0.638		
Self-efficacy in learning behaviors	SILB1	0.777	0.541	0.928
	SILB10	0.736		
	SILB11	0.769		
	SILB2	0.789		
	SILB3	0.654		
	SILB4	0.73		
	SILB5	0.726		
	SILB6	0.748		
	SILB7	0.691		
	SILB8	0.739		
	SILB9	0.719		

Specifically, the factor loadings for charismatic leadership range from 0.684 to 0.800, with an average variance extracted (AVE) of 0.547 and a composite reliability (CR) of 0.944. Pragmatic leadership shows factor loadings between 0.666 and 0.801, an AVE of 0.551, and a CR of 0.931. The factor loadings for ideological leadership range from 0.724 to 0.799, with an AVE of 0.592 and a CR of 0.853.

The three dimensions of learning engagement demonstrate strong psychometric properties. Behavioral engagement yields an AVE of 0.572 and a CR of 0.903; cognitive engagement shows an AVE of 0.600 and a CR of 0.900; and emotional engagement reports an AVE of 0.561 and a CR of 0.899, all meeting the recommended criteria.

Regarding academic self-efficacy, the factor loadings for learning ability self-efficacy range from 0.638 to 0.836 (AVE = 0.605, CR = 0.944), while the factor loadings for learning behavior self-efficacy range from 0.654 to 0.789 (AVE = 0.541, CR = 0.928).

Overall, the AVE values of all latent variables exceed the recommended threshold of 0.50, and all CR values are above 0.90, providing strong evidence of good convergent validity. These results confirm the internal consistency and construct validity of the measurement model.

This study employed AMOS 28.0 to examine the discriminant validity of the latent variables. As shown in Table 6, the results of the discriminant validity analysis indicate that all latent constructs demonstrate satisfactory discriminant validity, thereby confirming the adequacy of the measurement model. The Fornell–Larcker criterion was applied by comparing the square roots of the average variance extracted (AVE) with the inter-construct correlation coefficients. The square roots of the AVE values on the diagonal (charismatic leadership = 0.739, pragmatic leadership = 0.742, ideological leadership = 0.769, behavioral engagement = 0.757, cognitive engagement = 0.775, emotional engagement = 0.749, learning ability self-efficacy = 0.778, and learning behavior self-efficacy = 0.735) are all greater than the corresponding correlations between each construct and the others, satisfying the Fornell–Larcker criterion.

**TABLE 6:** Discriminant validity: Pearson correlations vs. square roots of AVE.

	Charismatic leadership	Pragmatic leadership	Ideological leadership	Behavioral engagement	Cognitive engagement	Emotional engagement	Self-efficacy in learning ability	Self-efficacy in learning behaviors
Charismatic leadership	0.739	0.742	0.769	0.757	0.775	0.749	0.778	0.735
Pragmatic leadership	0.538							
Ideological leadership	0.487	0.542						
Behavioral engagement	0.281	0.246	0.337					
Cognitive engagement	0.233	0.242	0.282	0.560				
Emotional engagement	0.255	0.246	0.273	0.564	0.579			
Self-efficacy in learning ability	0.210	0.211	0.247	0.292	0.234	0.238		
Self-efficacy in learning behaviors	0.182	0.197	0.196	0.318	0.234	0.240	0.572	

The inter-construct correlations are within acceptable ranges. Specifically, the correlations among the dimensions of teacher leadership range from 0.487 to 0.542; those among the dimensions of learning engagement range from 0.560 to 0.579; and the correlation between the two dimensions of academic self-efficacy is 0.572, all of which are lower than the square roots of their respective AVE values. Cross-construct correlations are relatively low: correlations between teacher leadership dimensions and learning engagement dimensions range from 0.233 to 0.337, while correlations between teacher leadership dimensions and academic self-efficacy dimensions range from 0.182 to 0.247. These results further demonstrate clear conceptual distinctions among the constructs and strong discriminant validity, providing a solid foundation for subsequent path analysis.

### 4.6 Structural equation modeling

**FIGURE 1:**
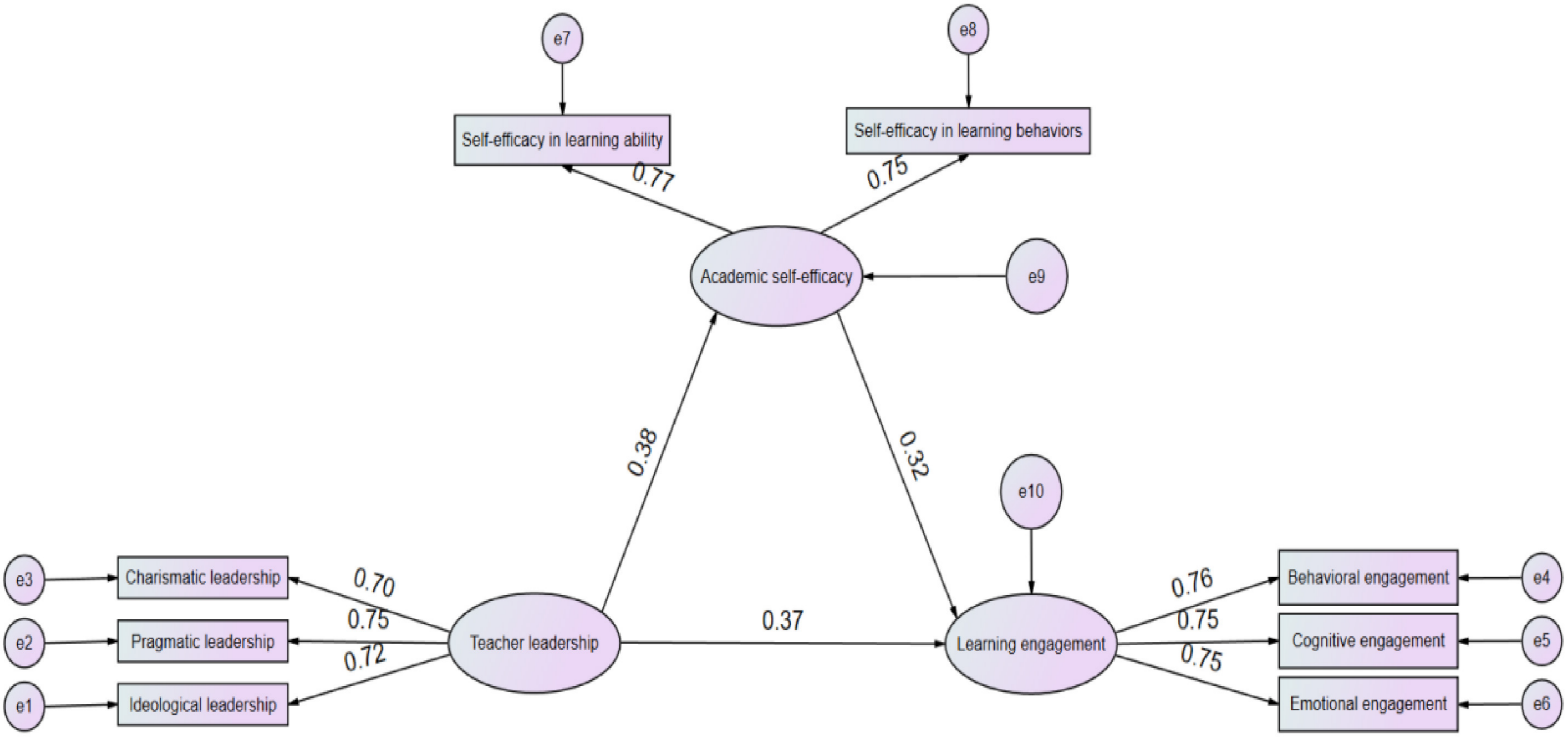
SEM path diagram.

This study employed AMOS 28.0 to construct a structural equation model (SEM) to test the proposed research hypotheses. The hypothesized model as shown in Figure 1 and Table 7 indicated a high degree of fit with the observed data, with all fit indices reaching excellent levels. The chi-square to degrees of freedom ratio (χ^2^/df = 1.227) was well below the strict threshold of 3, indicating an excellent model fit. For absolute fit indices, the GFI reached 0.987, approaching the perfect fit of 1.0; the RMR was 0.012, far below the critical value of 0.05, indicating minimal residuals; and the AGFI was 0.976, well above the recommended standard of 0.9. Incremental fit indices also performed outstandingly, with CFI = 0.994, NFI = 0.971, RFI = 0.954, TLI = 0.991, and IFI = 0.994, all approaching or exceeding the ideal level of 0.99. The excellent performance of all fit indices fully demonstrates that the structural equation model constructed using AMOS 28.0 accurately reflects the theoretical relationships among teacher leadership, learning engagement, and academic self-efficacy. The overall model fit is very high, providing a solid foundation for subsequent path coefficient testing and mediation analysis, and lending high credibility and scientific validity to the testing of the research hypotheses.

**TABLE 7:** SEM Fit indices.

Indicator	χ ^2^ */df*	GFI	RMR	CFI	NFI	RFI	TLI	AGFI	IFI
Reference value	< 3	> 0.9	< 0.05	> 0.9	> 0.9	> 0.9	> 0.9	> 0.9	> 0.9
Value	1.227	0.987	0.012	0.994	0.971	0.954	0.991	0.976	0.994

This study uses AMOS 28.0 to test the path coefficients, and the results of the structural equation model analysis in Table 8 showed that teacher leadership had a significant positive effect on academic self-efficacy (β = 0.382, B = 0.305, S.E. = 0.047, C.R. = 6.46, *p* < 0.001), and academic self-efficacy had a significant positive effect on learning engagement (β = 0.323, B = 0.326, S.E. = 0.060, C.R. = 5.453, *p* < 0.001). Teacher leadership also had a significant positive effect on learning engagement (β = 0.365, B = 0.294, S.E. = 0.046, C.R. = 6.325, *p* < 0.001). Regarding the control variables, neither gender (β = −0.011, *p* = 0.796) nor grade level (β = −0.036, *p* = 0.390) had a significant effect on learning engagement, whereas place of residence had a significant effect on learning engagement (β = −0.082, B = −0.078, S.E. = 0.039, C.R. = −1.984, *p* = 0.047). In summary, teacher leadership can directly promote students’ learning engagement and can also indirectly influence learning engagement by enhancing academic self-efficacy. This verifies the mediating role of academic self-efficacy in the relationship between teacher leadership and learning engagement.

**TABLE 8:** Path coefficients.

Path			Unstandardized coefficients	Standardized coefficients	S.E.	C.R.	*P*
Academic self-efficacy	< —	Teacher leadership	0.382	0.305	0.047	6.46	***
Learning engagement	< —	Academic self-efficacy	0.323	0.326	0.06	5.453	***
Learning engagement	< —	Teacher leadership	0.365	0.294	0.046	6.325	***
Learning engagement	< —	Gender	−0.011	−0.008	0.031	−0.258	0.796
Learning engagement	< —	Year	−0.036	−0.016	0.018	−0.859	0.39
Learning engagement	< —	Place of residence	−0.082	−0.078	0.039	−1.984	0.047

***p < 0.001.

### 4.7 Mediation effect analysis

Using AMOS 28.0, the mediating effect was tested via the Bootstrap method (5,000 resamples, 95% confidence interval). The results in Table 9 showed that academic self-efficacy had a significant mediating effect between teacher leadership and learning engagement [Estimate = 0.100, 95% CI (0.055, 0.166), *p* = 0.000]. Since the confidence interval does not include 0, this indicates that academic self-efficacy plays a significant mediating role in the pathway from teacher leadership to learning engagement.

**TABLE 9:** Mediation effect.

Path	Estimate	Lower	Upper	*P*
Teacher leadership– > Learning engagement– > Academic self-efficacy	0.100	0.055	0.166	0.000

## 5 Discussion and limitations

### 5.1 The relationships among teacher leadership, learning engagement, and academic self-efficacy

In this study, teacher leadership (three styles) has a significant positive impact on learning engagement, thus indicating that higher level of teacher leadership is associated with higher levels of learning engagement. This result aligns with Wang S. et al. (2024), which found that teacher leadership, especially in emotional and instructional aspects, plays a crucial role in increasing students’ learning engagement. Hunter J. P. et al. (2009) found that pragmatic leaders facilitate learning by setting clear objectives and breaking down complex tasks, making academic expectations more accessible and achievable, which boosts learning engagement. In a follow-up study, Hunter J. P. et al. (2011) highlighted how structured teaching approaches and effective feedback under pragmatic leadership promote content comprehension and a sense of academic involvement.

It was found that charismatic teachers stimulate students’ interest in learning, increase their involvement in classroom discussions and teamwork, and thereby enhance their pro-activeness and classroom engagement (Bolkan and Goodboy, 2011). Eyal et al. (2020) found that ideological leadership encourages students to maintain an active interest in their discipline beyond the classroom, thereby extending learning engagement.

Whether through personal charisma, the transmission of value-laden ideologies, or practical support, teachers’ leadership behaviors effectively enhanced the level of learning engagement among English major students. The reason may be that strong teacher leadership and different leadership style enhance students’ motivation, academic self-efficacy, and overall learning experience. Teachers who demonstrate leadership effectively create structured, interactive, and supportive learning environments, encouraging students to participate actively in their education. Furthermore, perceived teacher support reduces academic stress and improves students’ confidence, leading to higher levels of learning engagement.

This study also highlighted teacher leadership style (three styles) had a significant positive impact on the academic self-efficacy of the English major students, which suggested that when teachers exhibited personal charisma, conveyed positive educational values, or provided practical support and guidance in the learning process, they significantly enhanced English major students’ confidence in their academic abilities and their competence in completing academic tasks. This finding aligned with previous research. Charismatic teacher leadership emphasizes the teacher’s personal charisma, expressive motivation, and emotional contagion. Such teachers act as role models and transmit positive emotions, stimulating students’ intrinsic motivation and confidence in learning, thereby strengthening their belief in their ability to complete academic tasks (Conger and Kanungo, 1987). Pragmatic leadership emphasizes clear goals, instructional process management, and continuous feedback mechanisms. In the classroom, these teachers establish clear learning goals, provide phased guidance, and offer timely feedback, helping students form predictable and achievable learning paths, and a structured supportive environment helps students gradually accumulate successful experiences, which are an important source for enhancing self-efficacy (Li and Guo, 2018). Ideological leadership focuses on guiding students through the instillation of values, belief systems, and educational ideals. It emphasizes the social significance of learning and the mission of personal growth. Under this style, teachers’ leadership behaviors encourage students to integrate learning tasks with their self-identity, strengthen expectations for personal development, and thus enhance their academic self-efficacy (Wang and Li, 2020).

### 5.2 The mediating role of academic self-efficacy in the relationship between teacher leadership and learning engagement

This study showed that academic self-efficacy had a significant positive effect on learning engagement of English major students. And academic self-efficacy mediated the relationship between teacher leadership and learning engagement. This conclusion was consistent with previous research demonstrating that EFL learners with strong academic self-efficacy tend to display greater persistence, strategic learning behaviors, and a higher degree of engagement in language-related tasks. For example, studies have shown that academic self-efficacy positively correlates with EFL students’ motivation and autonomous learning, ultimately enhancing their language achievement (Li et al., 2024). Xu et al. (2021) pointed out that effectively enhancing students’ academic self-efficacy can significantly increase their learning engagement and bring about positive emotional experiences. Students’ SEF positively predicts their English academic performance (Xiong and Shen, 2016).

High self-efficacy increases motivation, resilience in the face of challenges, and willingness to invest effort—all of which enhance learning engagement. It also significantly influences the planning of learning tasks, psychological adjustments, and the choice of learning strategies for English language learners (Zhang, 2004). Li and Xu (2014) pointed out that students with high academic self-efficacy are more confident in their English language learning, tending to set higher learning goals, facing difficulties with a positive attitude and experiencing less anxiety, affecting their English language learning engagement. In this study, academic self-efficacy mediated the relationship between teacher leadership and learning engagement. This finding aligns with another research. A study among vocational students demonstrated that academic self-efficacy mediated the relationship between perceived teacher leadership and students’ deep learning engagement (Lu and Wang, 2025). Moreover, it was explored that academic self-efficacy mediates the relationship between teacher-student relationships and learning engagement for enhanced didactical outcomes (Wang X. et al., 2024).

### 5.3 Limitations

This study has several limitations that must be acknowledged to ensure a comprehensive understanding of its findings. First, this research was restricted to English major students from 3 colleges, thus limiting the generalizability of the results. Future research should include a diverse range of colleges and universities drawn from various regions and educational settings to ensure that the results of this research are applicable more broadly. Second, the study’s reliance on self-reported data introduces potential biases, such as social desirability bias and recall bias. Future studies could employ a mixed-methods approach, including qualitative interviews or focus groups, to obtain more factors that influence learning engagement and perceived social support. Furthermore, the cross-sectional nature of the study did not allow us to examine the longitudinal impacts of teacher leadership and academic self-efficacy on learning engagement of English major students. Future studies could adopt a longitudinal design to explore how teacher leadership and academic self-efficacy influence learning engagement over different stages of language education. Lastly, this study focuses on exploring the relationships among the three variables, without considering the inclusion of confounding variables. Future research should incorporate confounding variables to make the findings on learning engagement more reasonable, in-depth, and scientifically grounded.

## 6 Implications

Although the findings of this study were derived from a specific institutional context, which may limit their generalizability to other types of universities, the identified mechanisms linking teacher leadership to learning engagement provide a valuable foundation for broader institutional and policy-level initiatives. Expanding and adapting these practices across diverse educational settings may help validate and extend their applicability.

### 6.1 Enhancing teacher leadership by establishing institutional support and professional development mechanisms

This study demonstrates that teacher leadership has a significant positive impact on English major students’ learning engagement, suggesting that fostering strong teacher leadership is essential for enhancing student engagement and academic outcomes. To address this and ensure broader applicability, universities can leverage this finding by establishing institutional frameworks that support and develop teacher leadership. First, universities can implement a teacher leadership training system, regularly organizing workshops covering teaching management, classroom communication, and student psychological development to improve teachers’ professional competence and leadership skills (Dandan et al., 2025). Second, universities can establish a teacher development evaluation mechanism. By using student feedback, classroom observations, and peer reviews to evaluate teachers’ leadership behaviors and teaching performance, universities can incorporate these evaluations into promotion and annual performance reviews, thereby guiding teachers to focus on classroom leadership. Third, schools can build collaborative platforms by establishing “teacher leadership communities,” encouraging teachers to share teaching experiences and challenges, and promoting cross-grade and cross-disciplinary collaboration in teaching and research. Communication and collaboration among teachers are fundamental to the development of teacher leadership (Delcourt et al., 2024). While these initiatives hold great potential, their successful implementation may vary depending on the institutional context. Variations in resources, faculty engagement, and organizational readiness may influence the extent to which these strategies can be adopted across different universities. Therefore, universities should adapt these approaches to fit their unique needs and capabilities, ensuring that the benefits of teacher leadership development can be generalized to a broader range of educational settings.

### 6.2 Paying attention to students’ psychological needs and improve academic self-efficacy through differentiated instruction to enhance learning engagement

This study demonstrates that academic self-efficacy has a significant positive impact on English major students’ learning engagement, suggesting that teachers need to aim to improve students’ self-efficacy as one of their teaching goals, in order to strengthen intrinsic motivation and sustained learning engagement. First, progressive learning goals should be set to reinforce the sense of achievement. By gradually increasing the difficulty of tasks, students can gain a sense of accomplishment and build confidence through successful goal attainment. For example, in English writing courses, teachers can start with small-scale language exercises and gradually move toward complete text composition. Second, timely and positive feedback should be given to enhance students’ self-recognition. After completing tasks, teachers should offer specific and constructive comments such as “Your argument structure is clear, indicating that you understand the logic of paragraph development,” which helps reinforce a positive self-assessment (Li, 2024). Third, teachers should guide students to create personalized learning plans to cultivate autonomous learning. Teachers can assist students in setting phased learning goals based on individual strengths and weaknesses and regularly review and adjust their plans. This not only enhances self-efficacy but also contributes to long-term learning persistence. Fourth, peer support and learning role models should be encouraged. Research shows that observing role models and receiving peer support are important sources for developing academic self-efficacy (Lazarides and Warner, 2020). Teachers can organize group collaborative learning to guide students to learn from high-performing peers.

### 6.3 Providing professional psychological counseling services

This study demonstrates that academic self-efficacy has a significant positive impact on English major students’ learning engagement, and plays a mediating role between teacher leadership and learning engagement. English majors with low academic self-efficacy often struggle with language learning anxiety and lack of confidence, which can negatively impact their learning engagement and academic outcomes (Tang and Zhu, 2024). Research suggests that targeted psychological support can effectively enhance students’ self-efficacy and motivation (Wang and Zhan, 2022). English departments should establish accessible psychological counseling services that offer individual consultations and group support sessions to help students build self-confidence and cope with academic challenges. Regular anonymous surveys can be conducted to assess students’ psychological states and identify those with low self-efficacy for timely intervention. Additionally, integrating Mindfulness-Based Stress Reduction (MBSR) programs into the curriculum can contribute to improving students’ emotional regulation and promoting school attendance and school engagement (Sorrenti et al., 2025). Students’ sense of school connectedness can enhance their academic engagement, and this relationship is partly mediated by an increase in students’ mindfulness (Aghasaleh et al., 2024). Departments can also organize self-efficacy enhancement workshops, positive feedback mechanisms, and peer mentoring programs to promote a supportive learning environment and boost students’ engagement and confidence in English learning.

## 7 Conclusion

This survey of English major students revealed that teacher leadership was at a relatively high level, as were students’ academic self-efficacy and learning engagement. Both teacher leadership and academic self-efficacy had significant predictive effects on learning engagement, and academic self-efficacy mediated the relationship between teacher leadership and learning engagement. Therefore, enhancing the teacher leadership of English teachers and promoting students’ academic self-efficacy can effectively improve learning engagement among students and promote a positive academic atmosphere in language education of colleges and universities. This study provided comprehensive theoretical support and practical recommendations for foreign language teaching in high education.

## Data Availability

The original contributions presented in this study are included in this article/supplementary material, further inquiries can be directed to the corresponding authors.
